# Alcohol and Strychnine

**Published:** 1875-09

**Authors:** 


### ALCOHOL AND STRYCHNINE.

Mr. A. Young, formerly a resident of Brazil, expresses the opinion that the two poisons whose names head this paragraph, are antidotes for each other.

"A residence in Brazil for several years, where dogs are abundant and where, as a matter of necessity, hundreds of them are poisoned during the warm season by municipal decree—and that poison *strychnine*—gave me an abundant opportunity for experiment. I had previously been aware of the somewhat popular use of whisky for snake poison in our Southern States, and this I found not unknown in Brazil—any strong alcoholic spirit there subserving the same purpose.

"My first experiment was upon that of my servant man, who, enfeebled for a few days, had somehow stumbled upon a wine preparation, two grains strychnine to sixteen ounces of wine, taken it from my shelf and swallowed at least four ounces. A few minutes after he came to me with distressed feelings and immediate severe muscular contractions. With quick assistance he was laid upon his bed, and knowing nothing better I immediately prepared a mixture of two ounces spirits camphor and one pint of milk, which was with some difficulty swallowed owing to the severe contractions and contortions of the body. In five minutes' time he was calm, and at the end of an hour about his business.

"A few weeks afterward this same man was stung on his shoulder by a scorpion. A man more deathly pale, sick and helpless I never saw. This time he got rum and milk to the extent of a moderate drunk; soberness returned with his life.

"My experiments with dogs commenced with my own. I came in possession of a fine Florida bloodhound—one that had never forgotten his calling. His faithfulness by my fowl-yard prevented many hens and chickens seeking other quarters; but his enemies came at night and dosed him with strychnine, balls of meat being often thrown over the fence. Three times in as many weeks he was to all appearance hopelessly poisoned, but each time I saved him with powerful doses of spirits of camphor, with milk, and not only this dog, but many others, so many, indeed, that I was literally called "Douton Cao" (dog doctor.) I left Brazil at any rate a benefactor to good dogs. Within the last six months, I have successfully treated one case of strychnine poison with milk and spirits of camphor.

"The dose I would prescribe is 2 ounces spirits camphor with one pint of milk, and cause it to be taken as soon as possible. Await no time for emetics, and give none. If too much of the spirits and milk be taken it will be vomited up. If impossible to get it swallowed, inject *via ano*.

"I do not know that *the camphor* or the *milk*, one or both, is necessary. The real action of camphor is not known on the human system. I used it first from convenience, and, as it served well, continued to do so; the milk recommends itself as an excellent *vehicle* for its administration. After all, alcohol may do alone—but a stiff milk punch of spirits of camphor has proved the thing with me, and I would earnestly recommend it because it is always readily obtainable."

---

The physicians of Rutherford county, Tenn., having long enjoyed large but unremunerative practices, have resolved as follows: "That hereafter, before giving our professional attention to that class who have not heretofore paid their bills, or who are not clearly cases of charity or in some way extreme cases, we shall require of them either the cash in advance or an order from a responsible employer or landholder, making himself responsible for the payment of the bill or to have it otherwise secured."

---

A hot shovel held over varnished furniture will take out spots.

